# Occurrence of *Legionella* spp. in Man-Made Water Sources: Isolates Distribution and Phylogenetic Characterization in the Emilia-Romagna Region

**DOI:** 10.3390/pathogens10050552

**Published:** 2021-05-03

**Authors:** Marta Mazzotta, Silvano Salaris, Maria Rosaria Pascale, Luna Girolamini, Sandra Cristino

**Affiliations:** Department of Biological, Geological, and Environmental Sciences, University of Bologna, via San Giacomo 12, 40126 Bologna, Italy; marta.mazzotta2@unibo.it (M.M.); silvano.salaris@unibo.it (S.S.); mariarosaria.pascal2@unibo.it (M.R.P.); luna.girolamini2@unibo.it (L.G.)

**Keywords:** hospitals and communities, *Legionella* spp. distribution, agglutination test, *mip*-gene sequencing, phylogenetic analysis

## Abstract

*Legionella* species distribution in the Emilia-Romagna region, involving hospital (H) and community (C) environments, was conducted. *Legionella* culture, agglutination test, and *mip*-gene sequencing were applied on 240 isolates. The analysis showed a higher prevalence of non-*Legionella* *pneumophila* (n-*Lp*) species (84.1%) compared with *L. pneumophila* (*Lp*) (15.9%), with a higher frequency of n-*Lp* with respect to *Lp* species in both environments (77.6% and 96.4%, in H and C, respectively). The Shannon index showed a significant difference in *Legionella* distribution (*p* = 0.00017), with a significant abundance of *Lp* in the H compared with C environment (*p* = 0.00028). The continuous disinfection treatment in H could contribute to adaptive survival of the *Lp* species. Phylogenetic analysis revealed a conservative clade distribution between H and C: *L. feeleii* clade with three subclades in C and the *Lp* clade with five subclades in H and two in C, respectively. Our findings suggest the importance of *Legionella* surveillance both in H and C, with a focus on n-*Lp* species less connected to human disease. The *Legionella* prevalence and diversity found here indicate that geographical and temporal isolate evolution should be considered during surveillance, particularly in the light of global warming and changes in population risk factors.

## 1. Introduction

*Legionella* is a Gram-negative bacterium belonging to the *Legionellaceae* family associated with respiratory pathologies [1]. The family *Legionellaceae* consists of a single genus *Legionella* with more than 60 known species currently [2]. *Legionella* is widespread in natural aqueous environments and in man-made water systems such as potable water systems, cooling towers, air-conditioning units, and various water plumbing fixtures [3,4,5]. From these different sources, *Legionella* can be transmitted to humans through the inhalation of contaminated water aerosols. This results in the colonization of human lung alveolar macrophages, where the microorganisms replicate. *Legionella* infection is called legionellosis [6]. The term legionellosis commonly indicates both a mild flu-like illness, i.e., Pontiac fever, and a potentially fatal form of pneumonia, i.e., Legionnaires’ disease (LD) [4,7]. The risk of legionellosis is related to various factors, such as smoking, old age, and underlying diseases [8]. 

Although many *Legionella* species are considered potentially pathogenic to humans, the epidemiological data suggest that *Legionella pneumophila* (*Lp*) is the most common cause of LD. At present, 16 different serogroups (sgs) have been identified, and *Lp* sg1 accounts for over 90% of the cases worldwide [9,10]. In Europe, the number of reported LD cases increased by 65% between 2014 and 2018, with 85% of these cases attributed to *Lp* sg1. Of the 8974 cases with known outcomes, 8% were reported to be fatal. Most cases (72%) were community-acquired, 20% were travel-associated, 6% were associated with healthcare facilities, and 2% were associated with other settings [11].

Similarly, in Italy, in 2018, the incidence of legionellosis was 48.9 cases per million inhabitants, which was a significant increase compared with the previous year. In 99.9% of the reported cases, *Lp* was considered the agent responsible for the disease. The fatality recorded for the community and nosocomial cases is 10.9% and 51.7%, respectively [12]. 

These data are in contrast with the real environmental distribution of the genus *Legionella*, although standard serotyping of isolates is inadequate in epidemiological investigations, because *Lp* sg1 is considered predominant in several cases. The high incidence of human-reported disease caused by *Lp* sg1 is not due to its predominance in the environment, but is rather connected with the higher virulence of this strain; it is also influenced by the fact that the diagnostic technique is focused particularly on *Lp* sg1, as demonstrated by the urinary antigen test. At the time of reporting, it represented the first approach used in LD clinical diagnosis [3,13]. Further research is required to establish methods for the typing and classification of other potentially pathogenic *Legionella* species. The remaining *Lp* sgs identified include *Lp* sg3, sg6, and sg9, and especially non-*Legionella pneumophila* (n-*Lp*) species, such as *L. longbeachae, L. micdadei*, *L. bozemanii*, *L. feeleii, L. rubrilucens, L. londiniensis*, *L. anisa,* and *L. jordanis*; moreover, although these have already been described as etiological agents of *Legionella* diseases, with approximately 2–7% cases that can be attributed to them, the risk related to these species is underestimated [9,14,15,16]. However, the prevalence and geographical distribution vary greatly depending on the state or territory; for example, *L. longbeachae* constitutes 30.4% of the community-acquired *Legionella* isolates in Australia and New Zealand [4,10]. 

Several of the n-*Lp* species mentioned above are not well known, and some of them are less-studied, even though they are frequently isolated in the water distribution system; representing, in addition to *Lp,* a serious problem for public health [5,9,17]. To identify and trace strains causing legionellosis, it is important to correctly identify and type the *Legionella* strains in the patient, but even more in the environment, with a particular focus on elucidating the potential pathogenic role of n-*Lp* species. *Legionella* spp. environmental surveillance in generally performed in the presence of single, cluster, or epidemic events, other than as part of regular surveillance programs [18,19]. The subtyping of clinical and environmental isolates of *Legionella* is a powerful epidemiological tool for identifying clinical cases and linking them to environmental sources [20]. Cases of community-acquired legionellosis, for example, have been studied and linked to hotel water systems, where the risk may be linked to the intermittent use of water that is probably stagnated and has the consequent presence of biofilm forming microbes [21,22]. There is also evidence of the diffusion of *Legionella* in hot water distribution systems in private apartments and residences, spas, and swimming pools [23,24,25,26]. Many of these *Legionella* infections fall into the category of travel-associated diseases [27,28,29].

According to the scientific literature and the European and Italian Guidelines for the Prevention and Control of Legionellosis [18,19], the culture technique represents the “gold standard” method for the isolation of *Legionella* strains, followed by serological agglutination testing and molecular techniques, such as sequence-based typing (SBT) for the genotyping of *Lp* strains and *mip*-gene sequences for the genotyping of n-*Lp* species [30,31,32,33]. Unfortunately, these methods are used only during epidemiological investigations [32,34,35], and in routine *Legionella* environmental monitoring programs, as prescribed particularly for hospitals and healthcare facilities, they have not been applied. The test applied for *Legionella* identification includes serological methods such as the agglutination test, which is widely used by laboratories involved in clinical routine and environmental surveillance, with documented limits that lead to the misidentification or misagglutination of the circulating *Legionella* strains [36,37]. However, it can be asserted that in recent years, with the introduction of the new Italian Guidelines, the detection rate of environmental *Legionella* species has gradually increased in Italy.

The principal objective of this study was to assess the prevalence and distribution of *Legionella* spp. isolates from environmental water sources from several water distribution systems in the Emilia-Romagna region, Italy, as part of a *Legionella* environmental surveillance program. The water distribution systems were categorized into two main groups—hospitals (including hospitals, healthcare facilities, and long-term staying facilities) and communities (including spas, private apartments, bathhouses, companies, hotels, and gyms)—in order to compare the type of distribution and the phylogenetic relationship between the strains. Additionally, the molecular typing of *Legionella* spp. was conducted using *mip*-gene sequencing to assess the genetic diversity among the isolates. Our findings permit the elaboration of a map of *Legionella* distribution, to study the variability in terms of strain diversity between the two different environments. The focus on *Legionella* belonging to n-*Lp* species could improve the knowledge on less documented species in hospitals and communities that are less involved in surveillance programs. Moreover, the regional map of *Legionella* will help to control *Legionella* strain evolution (mutations, resistance, and pathogenicity) and, in the future, allow us to monitor and enforce preventive strategies with a focus on species evolution over time.

## 2. Results

### 2.1. Legionella Strains Environmental Distribution

All 240 isolates showed selective growth on *Legionella* Buffered Charcoal Yeast Extract (BCYE) cys+ agar. The agglutination test for *Legionella* identification showed that 38 (15.9%) isolates belonged to *Lp* species, all showing a positive agglutination reaction to sg1, and 202 (84.1%) isolates belonged to n-*Lp* species. Moreover, all of the isolates (*n* = 240) showed positive results in the *mip*-gene sequencing analysis, confirming the identification of *Legionella* spp. In particular, we identified several strains of *Lp* sg1: *Lp* sg1 strain Paris (11.7%), *Lp* sg1 strain Corby (1.7%), *Lp* sg1 strain Edelstein (1.3%), *Lp* sg1 strain Lens (0.8%), and *Lp* sg1 strain Alcoy (0.4%).

Within the n-*Lp*, several species were identified, with a high frequency of *L. taurinensis* (30.8%), followed by *L. anisa* (23.3%), *L. rubrilucens* (15.4%), *L. nautarum* (6.3%), *L. feeleii* (4.2%), *L. londiniensis* (3.3%), *L. jordanis* (0.4%), and *L. steelei* (0.4%). 

**Figure 1:**
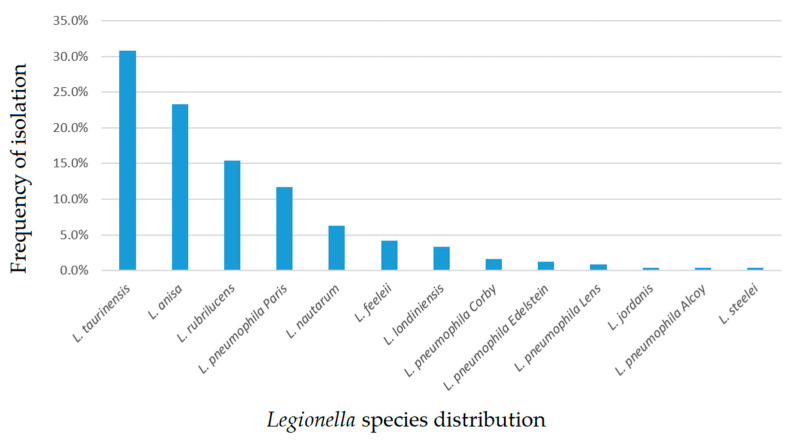
*Legionella* species frequency of environmental isolation in a water distribution system.

In Figure 1, we report the distribution frequency of the total *Legionella* spp. isolates.

**Figure 2:**
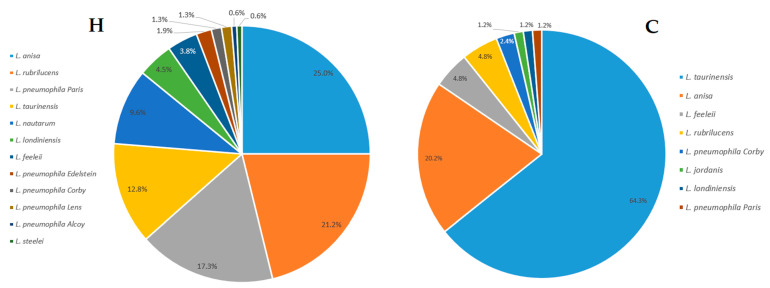
*Legionella* species distribution within hospital (H) and community (C) environments.

Using pie charts (Figure 2), we observed the type of strains and the relative frequency of isolation of *Legionella* isolates within hospitals (labeled as the H category) and communities (labeled as the C category), respectively. In general, the H category included 156 isolates and displayed a frequency of 77.6% and 22.4% for n-*Lp* species and *Lp* species, respectively. Similarly, in category C, we found 84 isolates, with a frequency of 96.4% and 3.6% for n-*Lp* species and *Lp* species, respectively.

### 2.2. Phylogenetic and Statistical Relationship

The genetic relationships among isolates were evaluated by aligning *mip*-gene sequences.

**Figure 3:**
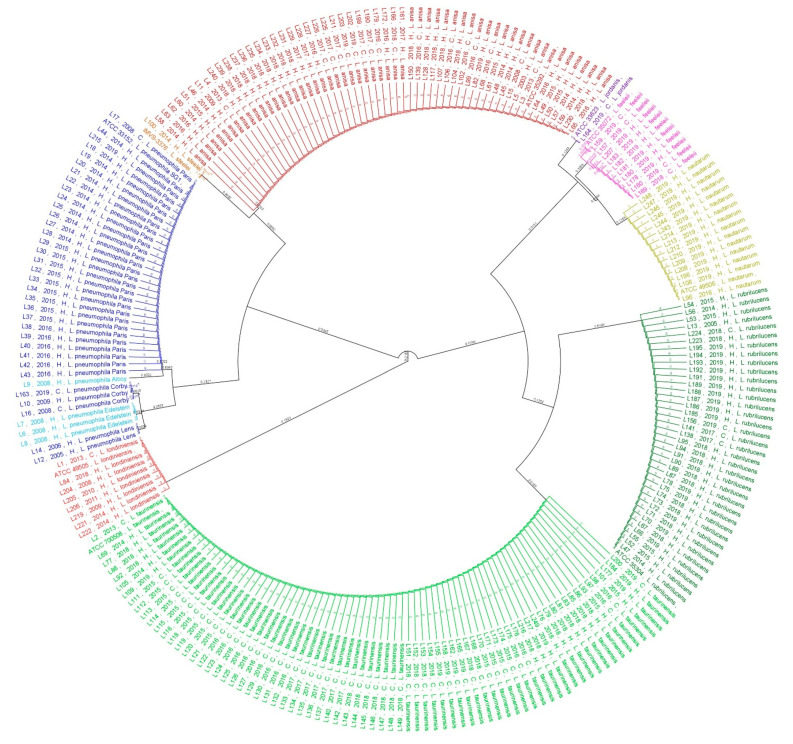
Phylogenetic tree based on the *Legionella mip*-gene sequences and relative reference strains. Branch labels represent values of substitutions per site.

A phylogenetic analysis was carried out on all 240 isolates and their respective American Type Culture Collection (ATCC) reference strains, as displayed in Figure 3.

**Figure 4:**
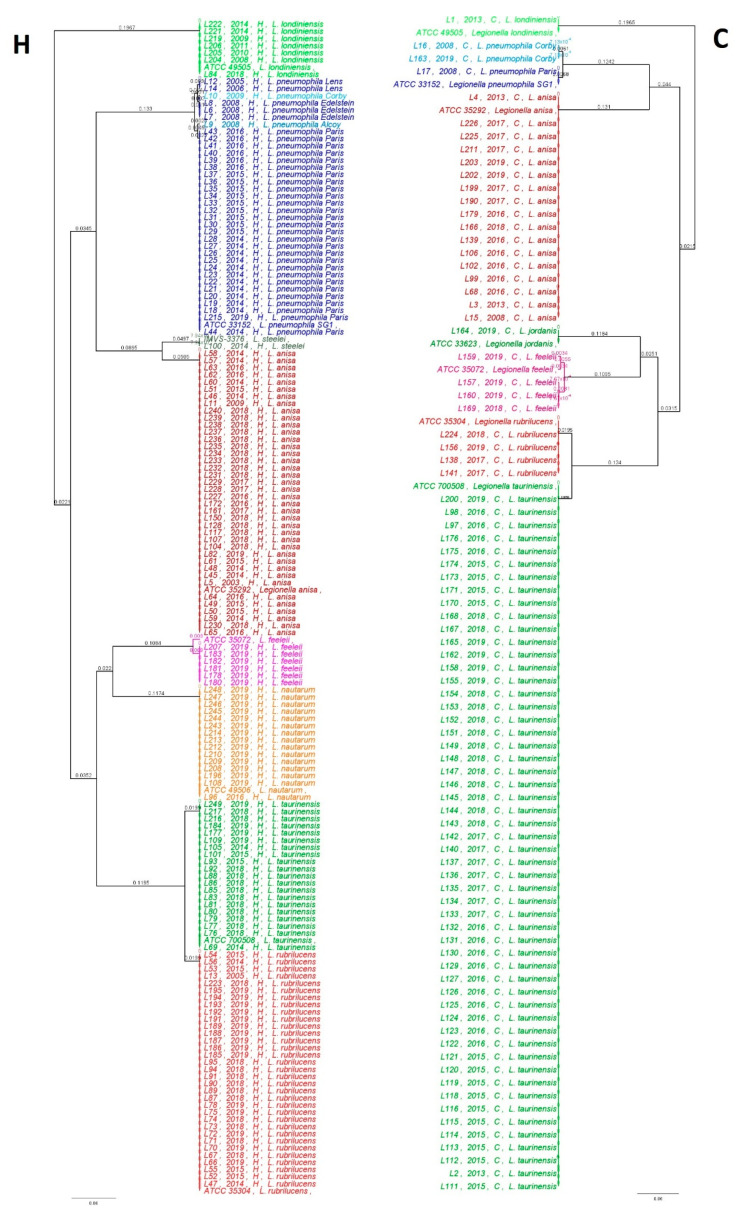
Phylogenetic relationship based on the *Legionella mip*-gene sequences collected from hospital (H) and community (C) environments.

An analysis of the population showed the presence of nine main clades represented by the following *Legionella* species: eight clades of *n*-*Lp* species represented by *L. steelei*, *L. anisa*, *L. jordanis*, *L. feeleii*, *L. nautarum, L. rubrilucens*, *L. taurinensis,* and *L. londiniensis,* and one clade represented by *Lp* sg1. Interestingly, five subclades were found within *Lp* sg1 represented by *Lp* sg1 strain Paris, strain Corby, strain Edelstein, strain Lens, and strain Alcoy; inside *L. feeleii*, we found three different subclades showing differences with respect to the ATCC 35072 reference strain. In particular, only one isolate of *L. feeleii* (labeled as L159) showed a match of 99.4% with the reference strain. In contrast, the L157 isolate showed ten mismatches (98.4%) and the remaining eight isolates, labeled as L207, L183, L182, L181, L180, L178, L160, and L169, showed 11 mismatches with the ATCC 35072 reference strain, with a percentage of similarity of 98.2%. The relationship between the genetic *Legionella* isolates in the two categories considered in this study (H vs. C) is shown in Figure 4.

The scale bar represents six substitutions/100 nucleotides in phylogenetic trees.

The genetic trees show how the two environmental categories were widely colonized by different *Legionella* species. In particular, the H and C environments displayed eight and seven different clades, respectively. In general, all strains belonging to the corresponding clade were conserved, and no genetic differences were found. An exception is the cluster of *Lp* sg1, as previously described. The H category displayed five clades related to different *Lp* sg1 strains Lens, Corby, Edelstein, Alcoy, and Paris, whereas in the C category, it was possible to discriminate only two *Lp* sg1 strains, Corby and Paris. The *L. feeleii* clade in both categories showed different subclades—three clades in C and one clade in H. In order to evaluate the diversity of the bacteria in H and C environmental categories, in terms of variety and total number of *Legionella* isolates (species) observed, the Shannon’s index (H’) test was performed. As shown in Table 1, the H’ values obtained were 1.98 and 1.14 for the H and C categories, respectively. The Hutcheson *t*-test showed a highly significant difference in terms of community composition of *Legionella* species (*p*-value *(p)* = 0.00017), in the two categories (H vs. C).

The analysis of the diversity in each environmental category was studied by comparing *Lp* and n-*Lp* populations. A χ^2^ test was carried out to investigate the diversity observed between categories, with significant differences regarding *Lp* distribution (*p* = 0.00028) (Table 2).

**Figure 5:**
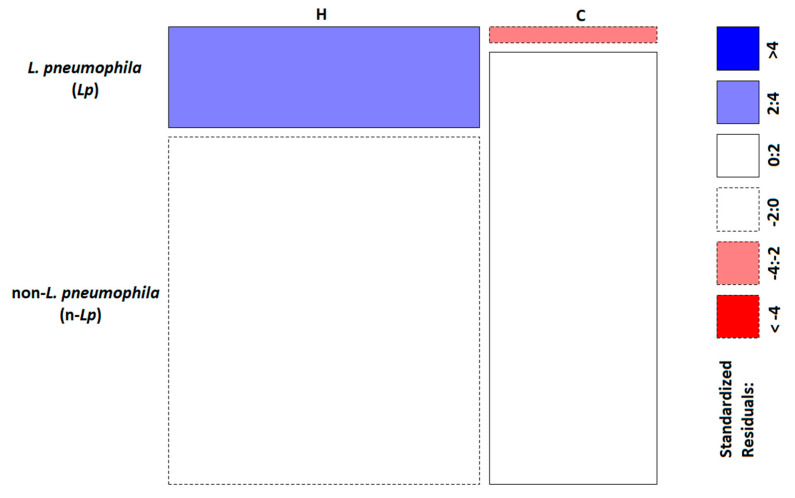
Pearson standardized residuals representation: blue indicates that the observed value is higher than the expected value if the data are random. Red indicates that the observed value is lower than the expected value if the data are random.

The graphical representation of Pearson residuals (Figure 5) indicated a significant association of *Lp* with the H category.

## 3. Discussion

Environmental *Legionella* surveillance is complicated, considering the diffusion of bacteria in the natural environment, often with a persistent rate in some geographical areas (e.g., *L. longbeachae*) [10,22,38]. The common outcome of environmental prevalence studies of *Legionella* spp. is the widespread diffusion of this microorganism in artificial water systems, despite the overall rarity of the most severe form of the disease. Moreover, a low level of environmental contamination does not guarantee the absence of clinical cases [39]. This is because the risk of contracting legionellosis is as a result of the environmental level of colonization, host receptivity, and pathogen factors (bacterial concentrations, virulence factors, and antimicrobial resistance). Being able to precisely detect the type of strain and assessing their characteristics so as to link them with the source of infection are the main objectives of this epidemiological investigation. Therefore, obtaining more information about both the clinical and environmental isolates during routine and epidemiological investigations could contribute to the refinement of the notification system of the disease and improve the diagnostic approach. Some of the difficulties in correctly identifying *Legionella* include the long culture time and the low isolation rate for some *Legionella* species; these species are not able to grow in the laboratory, but persist in environmental reservoirs in “non-culturable” forms [40,41,42].

The role of routine environmental monitoring, as suggested by the International and National Guidelines, remains the best approach to prevent epidemic events, such as improving the knowledge of circulating strains in the water distribution systems of a territory. This approach has been established considerably well in hospitals over the years, with the implementation of a risk assessment plan for *Legionella* prevention. However, in the community environments (e.g., apartments, companies, schools, swimming pools, and spas), this has not been applied yet. *Legionella* monitoring is undertaken after the notification of cases, as occurs during epidemiological investigations, or after the provision by Public Health Authorities, following the accommodation works, seasonal opening, or long closure, implementing a self-control plan.

The results reported in this study are in line with *Legionella* prevalence data regarding the wide colonization in both H and C water distribution systems [38]. Despite the high rate of cases attributed to *Lp* sg1, as reported by the Italian *Legionella* Epidemiological Annual Report [12] and several studies, our data show that in a hospital and community surveillance study, environmental predominance was related to n-*Lp* species, as described by other studies [43,44,45]. The analysis of *Legionella* frequency within the H and C categories showed that n-*Lp* are the major strains isolated in both categories: 77.6% in H and 96.4% in C, compared with *Lp* species that showed values of 22.4% in H and 3.6% in C. Interestingly, data were obtained for the n-*Lp* species found between the two categories. In H, the predominant strain was *L. anisa*, followed by *L. rubrilucens*, *L. taurinensis*, and *L. nautarum*, among others, most of which were less associated with human cases and are poorly understood from an ecological and pathogenic point of view. In C, the main strain identified was *L. taurinensis*, followed by *L. anisa*, *L. rubrilucens*, and *L. nautarum*. The presence of a high number of n-*Lp* species in both categories proves the importance of their correct identification and study, especially with respect to their potential pathogenic impact in the etiology of community-acquired pneumonia, as epidemiological data represent the primary source of information for *Legionella* infection [12]. Moreover, these findings suggest the role of implementing diagnostic studies, culture, serology, urinary antigen tests, and gene studies for *Legionella* species other than *Lp* species [46]. The study of diversity using Shannon’s index within H and C environments points out the differences in *Legionella* populations. The values of 1.98 found in the H category with respect to 1.14 in the C category show how the H environment contains significant *Legionella* population diversity, and in line with previous considerations, highlight that during environmental surveillance, one should consider not only the level of contamination, but also the *Legionella* species. 

The hospitals involved in this study had carried out a *Legionella* water safety plan, with a frequency of one to a maximum of four times/year, while the communities involved in this study were not routinely monitored—sometimes *Legionella* monitoring was performed only once a year, or when cases or clusters were reported. This difference in the frequency of monitoring, as in the *Legionella* surveillance program, could contribute to the reporting of incorrect data about the real prevalence of *Legionella,* especially with respect to communities. In addition, hospitals enforce a continuous disinfection treatment protocol to control bacterial proliferation, based on chemical agents well studied for *Legionella* treatment, such as chloride-dioxide (ClO_2_) and hydrogen peroxide/Ag+ (H_2_O_2_/Ag+). Both of them control *Legionella* contamination, but, as demonstrated in several studies, they show some limitations, especially with respect to the changes in the oxidative–reduction potential of water, with changes in the water chemistry/microbiology, as well as the oxidation and destabilization of inorganic contaminants that are released in water, with a significant health-safety impact [47,48]. Moreover, disinfection treatment often leads to a transient *Legionella* control with a rapid re-colonization of water distribution systems and the development of persistent *Legionella* strains that have become more tolerant to biocides, independent of the disinfection strategies applied [49,50,51]. These considerations are supported by the analyses of *Legionella* populations in each category, where the χ^2^ test revealed a higher association of *Lp* population in H than that in C, as was clearly evident from the Pearson residual analysis results. 

In contrast, although disinfection strategies are enforced to control *Legionella* in the community water distribution system, the presence of a highly diverse population in terms of the frequency of isolation and variety suggests their high potential for causing disease, as reported by the Italian *Legionella* Epidemiological Annual Report, where, in 2018, community-acquired cases represented 10.1% of the total cases. Considering that the domestic water distribution system in facilities such as spas, hotels, bathhouses, and gyms, involved in this study do not carry out systematic *Legionella* surveillance, this study demonstrates how the risk of exposure to *Legionella* is underestimated, especially in communities where people stay for a longer time for work or recreation activities. These findings suggest that understanding the ecology of *Legionella* populations in both H and C environments could aid water system management and disinfection strategies.

We believe that during surveillance programs, the study of the relationship among the strains has an important role in understanding the dynamics of contamination and evaluating the efficacy of the preventive strategies applied (e.g., disinfection treatment and maintenance measures). With this aim, a phylogenetic comparison among the *Legionella* isolates was performed. The analysis of data considering all 240 isolates, collected at different periods from the same sites (H or C), showed that both environments were colonized by *Legionella*, but interestingly, the species found were not only related to the common *Lp* species.

In the H environment, the *mip*-gene tree showed eight clades that were highly conserved, with exceptions in two species—*Lp*, where five subclades relating to the different strains were identified, and one clade represented by *L. feeleii* with 98.2% similarity to the ATCC 35072 reference strains, which could represent a misidentification. It is clear that this difference requires more research by studying more genes, as demonstrated in several studies [52,53,54,55]. Similarly, in the C population, it is possible to find seven clades, with two subclades in *Lp* (two different strains of Corby and Paris) and three in *L. feeleii,* with a percentage of similarity from 99.4% to 98.4%.

However, with the currently available data, we cannot assign this genetic diversity to spatial or temporal causes, considering that these isolates have been collected from different hospitals or communities located in the same region and isolated at different times. It is clear that these findings, in any case, could represent a first step in evaluating diversity during environmental surveillance. Our data demonstrate that the ecology and the population of *Legionella* species are more complex considering the incidence of disease (especially considering their high presence in the water distribution system) caused by a species that is poorly understood, and that this complexity could change their virulence or their pathogenic pathway in response to environmental stress, disinfection treatment, or in response to chemical-physical water characteristic changes [56,57]. The data reported are of great value, considering that *Legionella* has recently been mentioned in the new European Drinking Water Directive; it is included in the list of microbiological parameters to be evaluated for measuring water quality, even in the absence of cases [58]. 

Our study has some limitations. First, the study focused on the identification of *Legionella* using only the *mip*-gene sequencing approach [30,33]. Although the use of this approach has led to improved *Legionella* identification in comparison with the previous 16SrRNA gene approach, especially the identification of n-*Lp* species, in some cases, it can provide unsatisfactory results. This limitation is more evident when considering the isolates that exhibit differences within the same clade, which could lead to a probable misidentification of isolates; for example, the identification of different *L. feeleii* isolates belonging to the same clade could be improved by using an extensive approach such as whole genome sequencing. Second, the spread of bacteria in the environments monitored (H and C) does not permit the linking of geographical isolates, and prevents the study of geographical differences with respect to the water sources. In addition, the different *Legionella* surveillance programs limit the availability of data regarding the isolates coming from communities. 

This limitation could be overcome by increasing the routine monitoring of *Legionella* in different environments and by comparing the isolates collected by hospitals and communities over a longer period of time and in a geographical distribution that can better represent the regional environment (mountains, hills, and plains). It will be helpful to discover isolates with spatial-temporal variations in the region and better manage the risk associated with *Legionella* exposure within water distribution systems, especially in the context of global climate change, due to the high impact of global warming on the ecology and genotyping of microorganisms, as demonstrated for other microorganisms (e.g., *E. coli* and *Salmonella*) [38,59].

## 4. Materials and Methods

The sites involved in the study are located in the Emilia-Romagna region, and we focused especially on the Bologna province. These sites were chosen because according to Italian Guidelines [18]; some of these sites, which are hospitals and companies, have a specific *Legionella* monitoring program, while several other communities were occasionally monitored, for either a self-control plan or following their involvement in epidemiological investigation. According to their specific *Legionella* risk assessment plan, sampling was performed at different periods (e.g., years) for the same structures and/or after specific treatment (e.g., disinfection treatments). The names of the sites have been kept anonymous for privacy reasons; all of them have been classified into two main environmental categories: hospitals (H), which included hospitals (*n* = 95), healthcare facilities, and long-term staying (*n* = 61), and communities (C), which included spas (*n* = 5), private apartments (*n* = 35), bathhouses (*n* = 5), companies (*n* = 17), hotels (*n* = 19), and gyms (*n* = 3). Considering only the positive samples of the 240 isolates involved in this study, they were divided in 156 and 84 isolates for H and C, respectively.

### 4.1. Samples Collection and Microbiological Analysis

Two liters of hot water were sampled using 1-liter sterile polytetrafluoroethylene (PTFE) bottles containing sodium thiosulfate (20 mg/L), following the standard procedure suggested by the International Standard Organization (ISO) 19458:2006 [60]. Microbiological analysis was conducted within 24 h of environmental sampling. *Legionella* isolation was performed using a culture technique according to ISO 11731:2017 [61].

Two liters of hot water samples were processed with a membrane filtration technique using polyethersulfone membrane filters with a porosity of 0.22 μm (Sartorius, Bedford, MA, USA). Aliquots of 0.2 mL of untreated and 0.1 mL of concentrated, heated, and acid-treated samples were cultured on Glycine–Vancomycin–Polymyxin B-Cycloheximide (GVPC) plates (Thermo Fisher Diagnostic, Basingstoke, UK) and were subsequently incubated at 36 ± 1 °C with 2.5% CO_2_. The *Legionella* growth was evaluated every 2 days for 15 days. After the incubation period, colonies with morphologies associated with the *Legionella* genus were enumerated and five suspected colonies for each morphology, as indicated by ISO 11731:2017, were subcultured on Buffered Charcoal Yeast Extract (BCYE) agar with L-cysteine (cys+) and without L-cysteine (cys−) as a supplement, which is a selective medium used for *Legionella* isolation. The positive *Legionella* colonies were those that grew on the *Legionella* BCYE cys+ agar, but failed to grow on *Legionella* BCYE cys− agar. 

Isolated colonies grown on BCYE cys+ were serologically typed using an agglutination test (*Legionella* latex test kit, Thermo Fisher Diagnostic, Basingstoke, UK). The test allowed for the identification of *Lp* sg1, *Lp* sg2–14, and n-*Lp* species. The isolates identified as *Lp* sg2–14 were then processed for single serogroup identification using polyclonal latex reagents (Biolife, Milan, Italy).

Regarding the n-*Lp* species group, the pool of antibodies provided by the manufacturer recognizes only a few species commonly associated with clinical cases, such as *L. anisa*, *L. bozemanii* 1 and 2, *L. gormanii, L. longbeachae* 1 and 2, *L. dumoffii,* and *L. jordanis*; therefore, their identification was performed with a *mip*-gene sequencing analysis, as required by the European Working Group for *Legionella* Infections (EWGLI), according to the protocol described by Ratcliff et al. [30].

### 4.2. Molecular Analysis: DNA Extraction, PCR, and Mip-Gene Sequencing

*Legionella* DNA was purified and extracted using the InstaGene Purification Matrix (Bio-Rad, Hercules, CA). All of the isolates were processed and genotyped with *mip*-gene sequencing. Polymerase chain reaction (PCR) was performed using degenerate primers and was modified by M13 tailing to avoid noise in the DNA sequence. Specifically, *mip* amplicons (661–715 bp) were sequenced using M13 forward and reverse primers (*mip*-595R-M13R caggaaacagctatgaccCATATGCAAGACCTGAGGGAAC; *mip*-74F-M13F tgtaaaacgacggccagtGCTGCAACCGATGCCAC) to obtain complete coverage of the sequenced region of interest [62]. Gene amplification was carried out in a 50 μL reaction containing DreamTaq Green PCR Master Mix 2X (Thermo Fisher Diagnostic) and 40 picomoles of each primer; 100 ng of DNA extracted from the presumptive colonies of *Legionella* was added as a template. The same amounts of DNA from *Lp* type strain EUL00137, provided by EWGLI [63], and fetal bovine serum were used as positive and negative controls, respectively. Amplification was performed in a thermocycler under the following conditions: three min at 96 °C for pre-denaturation, followed by 35 cycles consisting of one min at 94 °C for denaturation, two min at 58 °C for annealing, and two min at 72 °C for extension, followed by five minutes at 72 °C for a final extension Then, the reaction mixtures were held at 4 °C.

The PCR products were visualized by electrophoresis on 2% agarose gel and were stained with ethidium bromide. Following purification, DNA was sequenced using BigDye Chemistry and was analyzed on an ABI PRISM 3100 Genetic Analyzer (Applied Biosystems, Foster City, CA, USA). Raw sequencing data were assembled using CLC Main Workbench 7.6.4 software (QIAGEN, Redwood City, CA, USA). The sequences were compared with those deposited in the *Legionella mip*-gene sequence database using a similarity analysis tool.

EWGLI has established an accessible web database (http://bioinformatics.phe.org.uk/cgi-bin/Legionella/mip/mip_id.cgi accessed on 16 February 2021) that contains sequence data from described species and allows for the identification of n-*Lp* species. Species-level identification was performed on the basis of ≥98% similarity to sequences in the database [33]. *Lp* sequences were identified at a strain level with a Basic Local Alignment Search Tool (BLAST) search [64]. The 240-nucleotide *mip* sequences generated for this study were submitted to the GenBank database. The provided accession numbers were as follows: MW524753-MW524817, MW021138, MW021138, MW052864, MW052865, MW052867, MW052869, MW052870, MW052873, MW052874, MW052876-MW052881, MW052883-MW052910, MW052912, MW052914, MW052915, MW052917-MW052922, MW052924-MW052942, MW052944, MW052953, MW052958-MW052972, MW052975-MW052977, MW052979-MW052994, MW052997-MW053005, and MW053007-MW053066.

### 4.3. Phylogenetic Analysis

Phylogenetic analyses were performed in Geneious Prime genome browser ["Geneious Prime 2020.2.4 (https://www.geneious.com accessed on 16 February 2021)"], over the 240 *mip*-gene sequences, both totally and individually in the H and C environmental categories. Multiple sequence alignments were carried out with MUSCLE v.3.8.425 [65], retaining the default settings. The phylogenetic trees were constructed using the Geneious Tree Builder, using Tamura-Nei [66] as a genetic distant model and the Unweighted Pair Group Method with Arithmetic mean (UPGMA) method [67] as a tree building method, and they were then bootstrapped using 100 replicates.

The reference strains for each *Legionella* species were included in the analysis, as follows: IMVS-3376 *L. steelei*, ATCC 35292 *L. anisa*, ATCC 33623 *L. jordanis*, ATCC 35072 *L. feeleii*, ATCC 49506 *L. nautarum*, ATCC 35304 *L. rubrilucens*, ATCC 700508 *L. taurinensis*, ATCC 49505 *L. londiniensis*, and ATCC 33152 *L. pneumophila*.

### 4.4. Statistical Analysis

Statistical analysis was performed using R version 4.0.2 (2020-06-22) (R Foundation for Statistical Computing, Vienna, Austria). The collected *Legionella* isolates were used to evaluate the species diversity in the two categories (H and C). The Shannon diversity index (H) [68] was used for this purpose, taking into consideration both the presence and absence of species and their abundance. The Hutcheson test was performed to calculate the significance of similarity between the H and C environmental categories. The Hutcheson test is a modified version of the classic Student’s t-test (*t*-test) that allows for a comparison of the diversity in two community samples using the Shannon diversity index [69]. A chi-square test (χ^2^) was used to determine a possible significant relationship between species and their habitats.

## 5. Conclusions

This study has demonstrated that both hospital and community environments are widely colonized by *Legionella* species, including the species most frequently to clinical cases and unknown relevant species. Communities are an important reservoir of *Legionella* and are often underestimated, whereas hospitals could be the best environment for *Lp* sg1. Moreover, our data suggest that the information on the relationship among the isolates over time and during the disinfection stages could be useful to understand the dynamics of contamination, changes in the *Legionella* population, and for the identification of effective corrective actions. 

Understanding the prevalence and distribution of *Legionella* not only in the hospital distribution system, but also in the domestic environment, should be the aim for future research in the light of the increasing amount of environmental (e.g., climate change) and population susceptibility (e.g., elderly people and underlying diseases) risk factors.

## Figures and Tables

**Table 1:** Evaluation of *Legionella* diversity between the hospital (H) and community (C) environmental categories according to the Shannon’s index (H’) and Hutcheson *t*-test.

*Legionella* Population	Hospital Category (H) No. of Isolates (*n*)	Community Category (C) No. of Isolates (*n*)
*L. anisa*	39	17
*L. feeleii*	6	4
*L. jordanis*	0	1
*L. londiniensis*	7	1
*L. nautarum*	15	0
*L. pneumophila (Lp)* Alcoy	1	0
*Lp* Corby	2	2
*Lp* Edelstein	3	0
*Lp* Lens	2	0
*Lp* Paris	27	1
*L. rubrilucens*	33	4
*L. steelei*	1	0
*L. taurinensis*	20	54
Shannon’s index (H’)	1.98	1.14
Hutcheson *t*-test	*p*-value (*p*) = 0.00017 *	

* Values are statistically significant at *p* ≤ 0.05.

**Table 2:** The analysis of *Legionella* species (*L. pneumophila* (*Lp*) vs. non-*L. pneumophila* (n-*Lp*) species) between hospitals (H) and communities (C) according to the *χ*^2^ test.

Environmental Category	*L. pneumophila* *(Lp)*	Non-*L. pneumophila* (n-*Lp*)
Hospitals (H)	35	121
Communities (C)	3	81
*χ*^2^ test	*p*-value *(p)* = 0.00028 *	

* Values are statistically significant at *p* ≤ 0.05.

## Data Availability

The data presented in this study are openly available in NCBI GenBank with the following accesion numbers: MW524753-MW524817, MW021138, MW052864, MW052865, MW052867, MW052869, MW052870, MW052873, MW052874, MW052876-MW052881, MW052883-MW052910, MW052912, MW052914, MW052915, MW052917-MW052922, MW052924-MW052942, MW052944, MW052953, MW052958-MW052972, MW052975-MW052977, MW052979-MW052994, MW052997-MW053005, and MW053007-MW053066.
